# The Correlation Between STONE Nephrolithometry Score and Hemoglobin Drop in Patients Undergoing Percutaneous Nephrolithotomy

**DOI:** 10.7759/cureus.11430

**Published:** 2020-11-10

**Authors:** Mohammad Shoaib, Muhibullah Bangash, Basit Salam, M Hammad Ather

**Affiliations:** 1 Surgery, Aga Khan University Hospital, Karachi, PAK; 2 Radiology, Aga Khan University Hospital, Karachi, PAK; 3 Urology, Aga Khan University Hospital, Karachi, PAK

**Keywords:** stone nephrolithometry score, hemoglobin (hemoglobin) drop, percutaneous nephrolithotomy (pcnl)

## Abstract

Objective

In this study, we aimed to determine the correlation between the STONE score [(S)ize of the stone, (T)opography or location, degree of (O)bstruction of the urinary system, (N)umber of stones, and (E)valuation of Hounsfield units] and postoperative hemoglobin drop in patients undergoing percutaneous nephrolithotomy (PCNL).

Methods

This was a prospective observational study and all adult patients aged 18-65 years undergoing unilateral, single-tract PCNL using 26 Ch. Amplatz sheath for renal calculi were included. The five variables of the STONE nephrolithometry score were calculated prior to the procedure. The stone-free rates were assessed on imaging at four weeks and complications were graded using the modified Clavien system.

Results

Of the 142 patients included, 75% were below 55 years of age. More than half of our patients were diabetic with more than 60% having a body mass index (BMI) above 25 kg/m^2^. The mean STONE score was 7 with 33% having a high (>9) STONE score. The mean hemoglobin drop was 1.15 +0.92 g/dL with eight patients (5.63%) requiring transfusion and one (0.7%) requiring angioembolization; one patient required readmission for observation. Complete STONE clearance was achieved with PCNL alone in 78.2% of the patients. There was a significant correlation of hemoglobin drop with the STONE score, stone size, and preoperative creatinine clearance. Patients with a hemoglobin drop of >1 g/dL had a higher STONE score and mean stone size. The overall complication rate was significantly higher (10.5%) in patients with a hemoglobin drop of >1 g/dL as compared to those with a hemoglobin drop of <1 g/dL (2.8%).

Conclusion

Stone complexity as measured by the STONE score correlates with post-PCNL hemoglobin drop, stone clearance, and complication rates. The STONE score may be used for preoperative counseling and to evaluate the potential need for transfusion.

## Introduction

Percutaneous nephrolithotomy (PCNL) is considered to be the standard treatment for moderate to large-sized renal calculi. It is minimally invasive and generally considered a safe and highly effective procedure. However, serious complications, although rare, are encountered in patients with a complex stone burden. Bleeding is common, although often minimal and self-limiting, and it can be life-threatening, requiring transfusions and angioembolization in rare cases. The need for transfusion varies from 1-11% [1-4], and up to 0.8% of the patients require angioembolization [5].

The predictors of postoperative bleeding include patient-related factors such as age [3], presence of diabetes mellitus (both type 1 and 2) [4], urinary tract infections [3], and preoperative hemoglobin levels, and stone-related factors such as large stones, staghorn calculi, stone location, the grade of hydronephrosis, and renal parenchymal thickness [6]. Perioperative factors like multiple access and prolonged surgery time can also influence postoperative bleeding [7].

Stone complexity is one of the factors that correlate with postoperative hemoglobin drop. One of the methods of measuring kidney stone complexity is the STONE score. The STONE score is calculated using five variables, abbreviated as the acronym ‘S.T.O.N.E.’. These include stone size, tract length (skin-to-stone distance), the degree of obstruction, the number of calyces involved, and stone essence (density) [8]. In their study, Said et al. showed that complete and partial staghorn calculi were associated with a greater blood loss compared to calyx stones [9]. They noted that stone complexity (Guy's Stone Score grades 3 and 4), preoperative creatinine level (≥1.4 mg/dl), intraoperative complication (perforation and extravasation), and duration of the operation (>83 minutes) were the most important factors associated with an increased risk of bleeding and transfusion during PCNL. In another study, Syahputra et al. [7] concluded that a larger stone burden or staghorn calculi required a greater amount of unit blood transfused during the PCNL procedure compared to smaller stones.

Stone complexity is a known predictor of postoperative bleeding after PCNL; however, whether the STONE score correlates with postoperative hemoglobin drop is not known. In this study, the correlation between the STONE score and postoperative hemoglobin drop was assessed among patients undergoing PCNL [10]. We hope our findings will help in counseling patients regarding transfusion requirement, hospital stay, and the possible need for embolization.

## Materials and methods

We performed this non-randomized study at the department of urology of a university hospital. After obtaining institutional review board and ethical review committee approval, all adult patients aged 18-65 years undergoing unilateral, single-tract PCNL using 26 Ch. Amplatz sheath for renal calculi were included in the study. We excluded patients undergoing additional procedures such as transurethral resection of the prostate/ureteroscopy (TURP/URS) along with PCNL; patients on anticoagulants, those with skeletal deformity, and those undergoing mini PCNL procedures were also excluded. Surgery was performed in a prone position under general anesthesia and the tract was dilated using serial metallic dilators. Stone fragmentation was done with an ultrasonic probe using EMS™ or Lithoclast® Master (EMS, Nyon, Switzerland). Smaller residual fragments were cleared using flexible nephroscope and laser. At the end of the procedure, a 12 Fr Foley catheter with the balloon inflated or a 12 Fr Nelaton catheter was placed as a nephrostomy tube at the discretion of the operating surgeon.

A senior radiologist (BS) at the workstation measured the variables such as stone size, tract length, degree and presence of obstruction (hydronephrosis), number of involved calyces, and stone essence (density). Each of the variables was scored according to the predefined system proposed by Okhunov et al. [8] and the STONE nephrolithometry score was calculated.

The sample size of 142 was calculated with an assumed correlation of 0.7 between the STONE score and hemoglobin levels, at a 5% level of significance, 80% power, and 10% inflation. The sample size was calculated by NCSS PASS software version 11 (NCSS, LLC, Kaysville, UT). For data collection, a non-probability continuous sampling technique was employed.

The primary outcome measure was postoperative hemoglobin drop, which was determined by calculating the difference between preoperative and postoperative hemoglobin measured on the first postoperative day after PCNL. Secondary outcomes were the number of blood transfusions required, the need for angioembolization within 30 days of the procedure, length of hospital stay, and 30-day complications according to modified Clavien grade.

Data were analyzed using SPSS Statistics version 25 (IBM, Armonk, NY). Continuous variables such as age, body mass index (BMI), and the STONE score were described in terms of mean/median and standard deviations. For post-hemoglobin drop, mean difference (preoperative and postoperative hemoglobin) was reported and assessed by paired t-test. Categorical variables such as gender were described in terms of frequencies and percentages. The correlation between the STONE score and postoperative hemoglobin drop was measured by the Pearson correlation/Spearman correlation coefficient as appropriate.

## Results

The basic demographics details are presented in Table 1. The STONE score and its individual parameters are shown in Table 2.

**Table 1:** Demographic details and baseline stone-related and clinical parameters SD: standard deviation; DM: diabetes mellitus; HTN: hypertension; BMI: body mass index

Parameters			Frequency (N=142)	Mean (±SD/%)
Age (years)				44.54 ±14.3
Gender	Male		87	61%
	Female		55	39%
Comorbidities	DM		75	52.81%
	HTN		80	56.30%
Hospital stay (days)				2.39 ±0.68
BMI, kg/m^2^	Total		142	27.26 ±5.45
	Underweight		8	5.60%
	Normal (BMI of 18-25)		44	30.90%
	Overweight		51	35.90%
	Obese		39	27.60%
Smoker			25	17.60%
Side of surgery	Left		79	55.20%
	Right		63	44.80%
Preoperative hemoglobin/hematocrit				13.45 ±2.0/41.62 ±5.47
Postoperative hemoglobin/hematocrit				12.30 ±1.97/37.70 ±5.20
Preoperative creatinine				1.00 ±0.38
History of stone surgery			48	34%
Postoperative double J stent			33	23%

**Table 2:** Correlations of the STONE scores and individual parameters with hemoglobin drop STONE: size of the stone, topography or location, degree of obstruction of the urinary system, number of stones, and evaluation of Hounsfield units; SD: standard deviation

Variables	Mean ±SD	Correlation coefficient	P-value
Total STONE score	7.56 ±1.64	0.16	0.05
Stone size (mm^2^)	381 ±200	0.49	0.03
Tract length (mm)	80.63 ±20.40	-0.09	0.26
Stone density (Hounsfield units)	910.3 ±358.9	0.001	0.99

Of the 142 patients included, 75% were below 55 years of age. More than half of our patients were diabetic with more than 60% having a BMI above 25 kg/m^2^. Almost all of our patients had normal preoperative hemoglobin (60% with a hemoglobin level of >13 g/dl). The mean STONE score was 7.12 with 33% having a high (>9) STONE score. This complexity is attributable to the presence of moderate/severe hydronephrosis and the involvement of more than three calyces in >50% of our patients. Overall mean hemoglobin drop was low (1.15 +0.92) in our patients with only eight (5.6%) requiring transfusion and only one (0.7%) requiring angioembolization. One of the patients required readmission and was managed conservatively with bed-rest. Complete STONE clearance was achieved with PCNL alone in 78.2% of the patients. Ancillary procedures for the residual stones were needed in 18 patients (12.7%) (Table 3).

**Table 3:** Outcome parameters including ancillary treatment for residual stones SD: standard deviation

Parameters		Number of patients	±SD/%
Mean hemoglobin/hematocrit drop			1.15 ±0.92/4.14 ±3.93
Blood transfusion		8	5.63%
Hematuria >24 hours		4	2.92%
Angioembolization		1	0.70%
Complications		19	13.38%
Readmission		1	0.70%
Fever		5	3.52%
Stone clearance		111	78.16%
Lost to follow-up		8	5.63%
Residual stone	Total	23	16.19%
	Shockwave lithotripsy	11	7.15%
	Ureteroscopy	6	4.22%
	Open stone surgery	1	0.70%
	Observation	5	4.12%

There was a significant correlation of hemoglobin drop with the total STONE score, stone size, and preoperative creatinine clearance (Table 4). Patients with a hemoglobin drop of >1 g/dl had a higher STONE score and stone size. Overall the complications were higher (10.5%) in patients with a hemoglobin drop of >1 g/dl as compared to those with a hemoglobin drop of <1 g/dL (2.8%) (Table 5).

**Table 4:** Stone-related parameters, calyces involved, and degree of obstruction

		Number of patients	±SD/%
STONE score	Mean		7.56 ±1.64
	Low (4-5)	19	13.38%
	Moderate (6-8)	75	52.82%
	High (>9)	48	33.80%
Stone size (mm^2^)	Mean		380.97 ±199.82
	0-399	91	64.08%
	400-799	46	32.39%
	800-1,599	5	3.53%
Tract length (mm)	Mean		80.63 ±20.40
	<100	115	81%
	>100	27	19%
Obstruction	No/mild	57	40.14%
	Moderate/severe	85	59.96%
Calyces involved (n)	1-2	61	42.15%
	3	36	25.35%
	Staghorn	45	31.70%
Stone density (Hounsfield units)	Mean		910.28 ±358.86
	<950	67	47.18%
	>950	75	52.82%

**Table 5:** Comparison of the groups regarding various outcome parameters in patients who had < or >1 gm/dl hemoglobin drop BMI: body mass index

	Hemoglobin drop of <1	Hemoglobin drop of >1	P-value
Female (n)	24	31	0.62
Males (n)	43	44	0.7
Age (years)	46.21	43.04	0.19
BMI	27.61	26.94	0.46
Preoperative hemoglobin (gm/dl)	13.19	13.7	0.14
Postoperative Hemoglobin (gm/dl)	12.73	11.93	0.02
Preoperative creatinine (mean, mg/dl)	1.04	0.97	0.28
Nondiabetic (n)	29	38	0.48
Diabetes present (n)	38	37	0.48
Nonhypertensive (n)	34	46	0.27
Hypertensive (n)	33	29	0.27
Non-smoker (n); smoker (n)	55; 12	57; 18	0.5
Left side (n)	39	40	0.68
Right side (n)	39	40	0.68
STONE score (mean)	7.12	8.03	0.04
Stone size (mm^2^)	219.81	468.71	0.03
Tract length (mm)	81.95	79.45	0.74

## Discussion

Hematuria is one of the most dreaded complications of PCNL. Mostly it is mild and self-limiting, however, occasionally it could be life-threatening. Ultrasound-guided access [10], miniaturization of the instruments [11], use of the balloon catheter nephrostomy tube [12], and tubeless PCNL with the use of tract sealant [13] are some of the ways described to minimize bleeding complications. Various patient and stone-related factors have also been studied to predict PCNL complications including previous interventions [14]. Various nephrolithometry scoring systems have been described to objectively quantify the parameters. In this study, we have attempted to correlate one of the most commonly used scoring systems, i.e., the STONE score, to predict bleeding complications of PCNL.

The correlation of the STONE score with postoperative hemoglobin drop has not been previously assessed in contemporary literature. Most of the previous studies have correlated the STONE score with estimated blood loss (EBL), which is often difficult to quantify. However, hemoglobin drop is a more objective way of assessing blood loss as compared to EBL.

Our study showed that there is a significant correlation between the STONE score and hemoglobin drop (p=0.05). Okhunov et al. [8] noted that the STONE score has a significant association with EBL (p=0.005) during PCNL. Another study by Yarimoglu et al. [15] showed no significant association between the STONE score and EBL. The mean hemoglobin drop was 1.15 ±0.92 in our study, which is similar to a recent study by Jamil et al. [12] comparing Foley and Nelaton™ catheter nephrostomy tubes. However, they noted no significant difference in the mean hemoglobin drop in the two groups with balloon versus non-balloon catheter nephrostomy respectively (1.08 ±0.7 and 1.14 ±0.69). Said et al. [9] noted a hemoglobin drop of 1.5 ±1 g/dl in the treatment of staghorn stones, which is comparatively higher than our hemoglobin drop; similarly, they noted a 14% blood transfusion rate as compared to only 5.63% in our study. Indication of a complex stone burden may be associated with greater blood loss. Hemoglobin drop of >1 g/dl was noted in patients with a STONE score of more than 8.03 as compared to a STONE score of less than 7.12 (p=0.04). Most patients with complications (15 out of 19), had a hemoglobin drop of >1 g/dl (p 0.04), which further implies that the increase in stone complexity shown by the STONE score is correlated with both the complications and blood loss. Our results showed that hemoglobin drop is also associated with stone size (p=0.03), which is similar to Okhunov et al.'s [8] study findings (p=0.047). Other individual components of the STONE score like skin-to-stone distance, stone density, and the presence of hydronephrosis did not show any significant association with hemoglobin drop in our study. This result is also similar to the findings of Okhunov et al. [8].

Another noteworthy observation in the current work is that a postoperative hemoglobin drop of >1 g/dl correlated with higher preoperative hemoglobin (13.7 g/dl) and lower preoperative creatinine (0.97 mg/dl). Hemoglobin drop was 1.76 g/dl with mean preoperative hemoglobin of 13.7 g/dl as compared to a drop of only 0.46 g/dl with mean preoperative hemoglobin of 13.2g/dl. This can be an incidental finding with a statistical significance and needs to be analyzed further in order to understand its clinical significance.

In our study, the mean hemoglobin drop was 1.15 ±0.92, and the mean hematocrit drop was 4.14 ±3.93. A previous study done by Zehri et al. [16] at the same institute showed a mean hemoglobin drop of 1.68 ±1.3 gm/dl, which indicates that the overall hemoglobin drop has decreased with time. Total blood transfusion in our current study has decreased to 5.63% (n=8) and only one patient (0.70%) required angioembolization. Zehri et al. [16] reported a higher transfusion rate of 14.2% (n=33) with one of the patients (0.43%) requiring angioembolization [16].

At the authors’ institution, all PCNLs are performed in the prone position and under general anesthesia. We predominantly use an ultrasonic probe for fragmentation or Lithoclast® Master with both ultrasound and pneumatic lithotripsy. The laser is not used for standard PCNL, except in a situation where a flexible nephroscope is used to overcome the difficulty to reach the caliceal fragment. Although regional and local anesthesia is also described for performing PCNL, in the current work, only general anesthesia was used. This, in our view, does not cause any limitations but limits the use of PCNL in patients who are ineligible for general anesthesia. However, it provides standardization in assessing outcomes in the current study.

The major strength of this study is that it is the first in-depth study correlating the STONE score with hemoglobin drop. We have included all types of patients, ranging from those with simple kidney stones to those with complicated ones. The major limitation of this study was that it was a monocentric study, with a relatively small sample size and short-term follow-ups. However, we believe that the results of this study will help in preoperative counseling of patients about the need for transfusions, hospital stay, and complications.

## Conclusions

Based on our findings, stone complexity as measured by the STONE score correlates with post-PCNL hemoglobin drop, stone clearance, and complications. Therefore, the STONE score can be used for preoperative counseling and to determine potential blood requirements.
